# Hippocampal amino‐3‐hydroxy‐5‐methyl‐4‐isoxazolepropionic acid GluA1 (AMPA GluA1) receptor subunit involves in learning and memory improvement following treatment with *Centella asiatica* extract in adolescent rats

**DOI:** 10.1002/brb3.1093

**Published:** 2018-08-14

**Authors:** Nor Aqilah Binti Mohd Yusuf Yeo, Sangu Muthuraju, Jia Hui Wong, Faruque Reza Mohammed, Mohd Harizal Senik, Jingli Zhang, Siti Rafidah Yusof, Hasnan Jaafar, Mohd llham Adenan, Habsah Mohamad, Tengku Sifzizul Tengku Muhammad, Jafri Malin Abdullah

**Affiliations:** ^1^ Center for Neuroscience Services and Research(P3Neuro) Universiti Sains Malaysia Jalan Hospital Universiti Sains Malaysia Kota Bharu Kelantan Malaysia; ^2^ Department of Neurosciences School of Medical Sciences Universiti Sains Malaysia Jalan Hospital Universiti Sains Malaysia Kota Bharu Kelantan Malaysia; ^3^ Department of Neurosciences Hospital Universiti Sains Malaysia Jalan Hospital USM Kota Bharu Kelantan Malaysia; ^4^ Centre for Drug Research Universiti Sains Malaysia Penang Malaysia; ^5^ Department of Pathology School of Medical Sciences Universiti Sains Malaysia Jalan Hospital Universiti Sains Malaysia Kota Bharu Kelantan Malaysia; ^6^ Faculty of Applied Sciences Universiti Teknologi MARA Shah Alam Selangor Malaysia; ^7^ Institute of Marine Biotechnology Universiti Malaysia Terengganu Terengganu Malaysia

**Keywords:** adolescent, AMPA receptor, *Centella asiatica*, GABA receptor, hippocampus, learning and memory

## Abstract

**Introduction:**

*Centella asiatica* is an herbal plant that contains phytochemicals that are widely believed to have positive effects on cognitive function. The adolescent stage is a critical development period for the maturation of brain processes that encompass changes in physical and psychological systems. However, the effect of *C. asiatica* has not been extensively studied in adolescents. The aim of this study was therefore to investigate the effects of a *C. asiatica* extract on the enhancement of learning and memory in adolescent rats.

**Methods:**

The locomotor activity, learning, and memory were assessed by using open field test and water T‐maze test. This study also examined changes in neuronal cell morphology using cresyl violet and apoptosis staining. We also performed immunohistochemical study to analyse the expression of the glutamate AMPA receptor (α‐amino‐3‐hydroxy‐5‐methyl‐4‐isoxazolepropionic acid) GluA1 subunit and the GABA receptor (γ‐Aminobutyric Acid) subtype GABA_A_ α1 subunit in the hippocampus of the same animals.

**Results:**

We found no significant changes in locomotor activity (*p *>* *0.05). The water T‐maze data showed that 30 mg/kg dose significantly (*p *<* *0.05) improved learning, memory, and the memory consolidation phase but had no effect on reversal learning (*p *>* *0.05). Histological data revealed no neuronal morphological changes. Immunohistochemical analysis revealed increased expression of the AMPA GluA1 receptor subunit but there was no effect on GABA_A_ receptor α1 subunit expression in the CA1 and CA2 subregions of the hippocampus.

**Conclusions:**

The *C. asiatica* extract therefore improved hippocampus‐dependent spatial learning and memory in a dose‐dependent manner in rats through the GluA1‐containing AMPA receptor in the CA1 and CA2 sub regions of the hippocampus.

## 1 INTRODUCTION

*Centella asiatica* is one of many botanical plants that contain beneficial phytochemicals. It is a tropical plant from the Apiaceae family and is widely distributed in Southeast Asian countries, such as India, China, Sri Lanka, Malaysia, and Indonesia, as well as South Africa and Madagascar (Orhan, 2012). The major chemical constituents found in *C. asiatica* are triterpenoid derivatives, such as asiatic acid, madecassic acid, asiaticoside, and flavonoids (Saoji et al., 2016; Sari et al., 2014; Vasavi, Arun, & Rekha, 2016). This plant has been widely used in Ayurvedic, African, and Chinese traditional medicine (Giribabu, Srinivasarao, Swapna Rekha, Muniandy, & Salleh, 2014; Vasavi et al., 2016). The best known beneficial effects of *C. asiatica* are its neuroprotective effect and its effects on cognitive function, particularly learning and memory (Doknark, Mingmalairak, Vattanajun, Tantisira, & Tantisira, 2014; Giribabu et al., 2014; Gray, Harris, Quinn, & Soumyanath, 2016; Gray et al., 2017; Sirichoat et al., 2015).

Learning is the process of acquiring new knowledge about the world and environment, whereas memory is the process of storage or retention of the acquired knowledge. Memory consists of an individual's ability to record sensory stimuli, events, and information, as well as to retain this information over a short or long period of time and recall it when needed (Chakravarthi & Avadhani, 2013). Learning and memory processes involve several regions of the brain including the cortex, amygdala, cerebellum, and hippocampus. A series of processes take place from memory formation to memory recall that include encoding, memory storage, consolidation, and recall. The mechanism of memory formation involves induction of long term potentiation (LTP) and expression of synaptic plasticity in which the hippocampus is activated while processing occurs (Yolanda, Sari, Rochmah, & Suharmi, 2015). The hippocampus plays important role in spatial memory, navigation, and long‐term memory (Chakravarthi & Avadhani, 2013; Shen, Sabaliauskas, Yang, Aoki, & Smith, 2017). It is a major component of the brain and consists of main subregions CA1, CA2, CA3, and DG areas which are composed of tightly packed pyramidal neurons (Cherubini & Miles, 2015).

Two types of brain cells are involved in learning and memory: neurons and glial cells. Neurons communicate with others cells by the release of chemical neurotransmitters, which act transiently on postsynaptic receptors (Fields et al., 2014; Krebs, Weinberg, & Akesson, 2012; Nuss, 2015). Several neurotransmitters are located in the brain, but glutamate is the major excitatory neurotransmitter. Glutamate is involved in almost all functions of the central nervous system (CNS), especially in the cortical and hippocampal regions, whereby 70% of all excitatory synapses in the central nervous system utilize glutamate as a neurotransmitter (Danysz & Parsons, 2012). The major inhibitory neurotransmitter in the CNS is γ‐aminobutyric acid (GABA), which has been estimated to function in at least one‐third of all CNS neurons as the primary neurotransmitter (Nuss, 2015).

Long‐term potentiation (LTP) is a major cellular mechanism underlying learning and memory. The involvement of LTP in the hippocampus requires the activation of two key ionotropic glutamate receptors: α‐amino‐3‐hydroxyl‐5‐methyl‐4‐isoxazolepropionic acid (AMPA) and N‐methyl‐D‐aspartate (NMDA) receptors at glutamatergic synapses (Pandey, Singh, & Prasad, 2015). Glutamate is one of the most important excitatory neurotransmitters in the CNS where it generally mediates fast excitatory transmission across the nervous system and functions in various integrative brain functions, as well as in the development of the brain (Mukherjee & Manahan‐Vaughan, 2013). The inhibitory neurotransmitter γ‐aminobutyric acid (GABA) also plays a role as a regulator of learning, memory, and synaptic plasticity (Wang et al., 2012).

This study aimed to investigate the effect of the extract of *Centella asiatica* on learning and memory in adolescent rats. It is suggested that early postnatal stage of rodent is a vulnerable period in brain development and treatment with external agent during this period can bring about significant changes to the cognitive behavior. At birth, the brain is very immature and it continues to develop from intrauterine life and proceeds well after birth and even adolescence. Adolescence is a critical period transitioning between childhood and adulthood, which is important for neurodevelopment that characterized by various changes in anatomy and physiology of the brains (Li, Masugi‐Tokita, Takanami, Yamada, & Kawata, 2012) as well as development of behavioral and biological systems (Hsu et al., 2015), cognitive, psychological, and maturation of neurotransmitter system (Arain et al., 2013; Mengler et al., 2014; Schneider, 2013). In rodents, the term ‘adolescence’ represents the entire postnatal period, which ranges from approximately a week after weaning period, at postnatal day 28 (PND 28), to as late as postnatal day 60 (PND 60) (Hammerslag & Gulley, 2014; Mengler et al., 2014). During the transition period in adolescence, rodent's hippocampus and prefrontal cortex undergo significant development with learning and memory continue to develop throughout the adolescence period (Uysal et al., 2015). Moreover, during adolescence, drastic changes of neuronal architecture and function occur that concomitantly lead to distinct behavioral alteration (Arain et al., 2013; Mengler et al., 2014; Schneider, 2013). Thus, during this critical period of growth and development, the cells can be easily influenced by the nutrition intake (Beheshti, Hosseini, Vafaee, Shafei, & Soukhtanloo, 2016) and any nutritional nourishment in this stage can have beneficial effects to the brain.

## 2 MATERIALS AND METHODS

### 2.1 *Centella asiatica* extract

The extract of *C. asiatica* (Reference number: AuRins‐MIA‐1‐0) provided by Universiti Teknologi MARA (UiTM) to USM originated from the dried whole plant and was extracted into a brown powder form by ethanolic extraction.

### 2.2 Animals

The procedures were performed on 28 albino male Wistar rats (1–2 months of age) with an average body weight of 100–150 g, obtained from Alchemy Supplies Sdn. Bhd. (Selangor, Malaysia) and kept at the Laboratory Animal Research Unit, School of Medical Sciences, Universiti Sains Malaysia (USM), Kelantan, Malaysia. Animal ethics has been approved by Universiti Sains Malaysia with ethics approval number: USM/Animal Ethics Approval/2015/(98)699). All rats were kept in polypropylene cages (421 mm × 290 mm × 190 mm), exposed to 12 hr light/dark cycles and supplied with a pellet diet (Gold Coin 702P, Gold Coin Feedmills Sdn Bhd, Port Klang, Malaysia) and water ad libitum. Pine shavings (Living World 61233, Rolf C. Hagen, Inc., Holland) were used as bedding material and changed at 2‐day intervals.

### 2.3 Groups

A total of 28 rats were divided into three experimental groups. Group 1 (*n* = 8) served as a control group and received 0.5 ml double distilled water. Group 2 served as one treatment group (*n* = 10) and received 30 mg/kg of *C. asiatica* extract. Group 3 served as the other treatment group (*n* = 10) and received 300 mg/kg of *C. asiatica* extract. The extract was administered to the rats daily by oral gavage for two consecutive weeks (14 days). Their locomotor activity behaviors and learning and memory performance were then assessed and subsequently they were sacrificed for morphological studies.

### 2.4 Open field test (OFT)

The apparatus consisted of a Perspex cage (height: 40 cm, length: 90 cm; width: 90 cm), with the bottom divided into 25 small squares (18 cm × 18 cm). A video camera was placed 250 cm above the open field to record the trials (ArcSoft TotalMedia 3.5, ArcSoft, US). The experiment started with each rat placed in the center of the open field and the locomotor activity was digitally recorded for 5 min. The open field floor was wiped with 30% ethanol between trials and allowed to dry before the next trial. The number of lines crossed was manually counted offline for each rat (Salihu et al., 2016).

### 2.5 Water T‐maze test

This modified method of the water T‐maze used was designed and custom made by Dr. Jingli Zhang and constructed from Perspex material made by Bengkel Plastik Berek 12, Kelantan, Malaysia. Each arm of the water T‐maze was 50 cm long, 20 cm wide, and 50 cm high. The water T‐maze was 90 cm long and 60 cm wide. The water T‐maze was then filled with 23°C (±1°C) water to a depth of 22 cm, which was 2 cm above the surface of the platform. The platform was a 7 cm × 7 cm × 22 cm rectangle made from Plexiglas to fit specifically into the water T‐maze (Guariglia & Chadman, 2013; Locchi, Dall'Olio, Gandolfi, & Rimondini, 2007). Each experiment started with position habit acquisition learning, in which the rats were placed into the water T‐maze without a platform and were allowed to swim for 60 seconds for pretraining, as a habituation step. On the training days, the platform was assigned in the arm opposite to the first arm selected during pre‐training (the platform was placed at the left side). The rats were given eight training trials per day, dried with a towel, and allowed to rest in between trials for the amount of time it took for all rats in the cohort to completed their trial (approximately 7–10 min). At the beginning of each trial, the rats were placed into the pool at the starting arm of the T, and they were allowed up to 60 s to find the location of the platform. These trials were conducted for five consecutive days, and then they began probe trials for the memory consolidation test without a platform, followed by training for reversal position learning. The reversal learning trials used the same procedure as for learning acquisition, but the platform was switched to the opposite arm of the T (the right side) and reversal learning was conducted for three consecutive days. Rats were placed into the pool at the starting arm of the T and were expected to find the new location of the platform. Each rat was given eight trials per day. Rats were then dried with towel and allowed to rest for 7–10 min in between trials. The entire test was recorded by video camera (ArcSoft TotalMedia 3.5, ArcSoft, US). The escape latency in seconds was recorded and analyzed.

### 2.6 Histological analysis

For tissue collection and processing, the rats were euthanized with mild anesthesia (sodium pentobarbital; 0.27 ml) (Dorminal, DIN 02333708, Alfasan, Woerden, Holland) and perfused intracardially with 0.1 M phosphate‐buffered saline (PBS, pH 7.0) for 2 min, followed by 4% paraformaldehyde (PFA, pH 7.0) (Acros Organics, AC416780010, Fisher Scientific, USA) for prefixation of the tissues for 3 min. The brain was carefully dissected out, postfixed in 4% paraformaldehyde and kept in a refrigerator at 4°C at the Centre Research Laboratory (CRL), USM, Kubang Kerian Malaysia. Tissues were then processed for paraffin‐embedded sectioning according to the procedure described by Salihu et al. (2016).

### 2.7 Cresyl violet staining

Paraffin‐embedded sections were deparaffinized and rehydrated with a gradient of alcohol and washed with tap water. Tissue sections were stained with cresyl violet staining solution (C5042, Sigma Aldrich, USA) and washed with tap water. The tissues were then dehydrated in a gradient of alcohol, cleared with xylene (C0900‐2190239, HmbG Inc., Germany), and mounted in DPX mounting medium (360294H, BDH Chemicals, UK). Tissue sections were covered with coverslips (G0543, HmbG Inc. Germany) and observed and analyzed with a light microscope with an image analyzer (20 × objective lens power) (Olympus, BX‐14‐32PO2, Olympus Corporation, Japan) by three blinded investigators (Al‐Rahbi et al., 2014; Moorthi, Premkumar, Priyanka, Jayachandran, & Anusuyadevi, 2015).

### 2.8 Annexin V‐FITC apoptosis detection kit/propidium iodide assay

Annexin V‐FITC propidium iodide staining assay and fluorescence microscopy were employed to identify cells undergoing apoptosis. The annexin method was performed using a TACS Annexin V‐FITC apoptosis detection kit (4830‐01‐K, Trevigen, Inc., US), following the manufacturer's instructions. Briefly, paraffin‐embedded sections were deparaffinized and rehydrated with a gradient of alcohol and washed in PBS. The slides were then treated with blocking solution and incubated for 20 min in the dark. After the incubation, the slides were carefully wiped around the samples and washed in PBS. A 25 μl of buffer was added and the samples were incubated for 10 min. Next, 25 μL of Annexin V incubation reagent were added to the slide for 30–45 min, followed by washing with PBS. The slides were then mounted using fluorescent mounting medium (Prolong Gold Antifade Mountant, Molecular Probes, P10144, ThermoFisher Scientific, USA), covered with coverslips and viewed immediately with a fluorescence microscope with an image analyzer (Olympus, BX‐14‐32PO2, Olympus Corporation, Japan) under a green filter (20 ×  objective lens power) by three blinded investigators.

### 2.9 Immunohistochemical staining

Immunohistochemical staining was conducted using the HRP/DAB technique. Rabbit specific HRP/DAB detection IHC kit (AB64261, Abcam, UK) with labeled streptavidin‐biotin immunoenzymatic antigen detection system that includes chromogen 3,3′diaminobenzidine (DAB) was used for detection and counterstained with hematoxylin. Tissue sections were deparaffinized, rehydrated, and rinsed in 0.1 M PBS buffer, followed by incubation with hydrogen peroxide blocking solution (AB64218, Abcam, UK) for 10 min at room temperature in dark place. Protein blocking solution (AB64226, Abcam, UK) was applied on the tissue sections for 5 min at room temperature to block nonspecific background staining and washed with PBS buffer. The sections were incubated overnight at 4°C with 70 μl of rabbit polyclonal primary antibodies to the GABA_A_ receptor α1 (AB33299, Abcam, UK) diluted at 1:100 using antibody diluent (50809, Dako, Denmark). Another set of tissue slides was similarly tested for rabbit monoclonal antibodies to glutamate receptor 1 (AMPA subtype) (AB183797, Abcam, UK) at 1:100 dilution. Following incubation of primary antibodies, secondary antibodies containing biotinylated goat anti‐rabbit were applied onto tissue sections and incubated for 1 hr at room temperature, followed by 15 min of incubation with streptavidin peroxidase. All tissue sections were rinsed with PBS in between incubation steps. The tissue sections were incubated with DAB staining for 5 min and rinsed with distilled water. A drop of Harris Hematoxylin stain (Labstain, N082/816, Labchem Sdn. Bhd., Malaysia) was then applied and the tissue was counterstained for 1 min. The slides were rinsed with distilled water, mounted with Faramount aqueous mounting medium (5302580, Dako, Denmark), and covered with a coverslip. This experiment was replicated three times using the same procedure. The staining was observed with a light microscope with an image analyzer (Olympus BX‐14‐32PO2, Olympus Corporation, Japan) with a 4 × magnification field. Three sections were used for immunohistochemical scoring of each sample. The expression intensity was observed and analyzed qualitatively and assessed by three blinded investigators using the following subjective scale: mild (+), moderate (++), and strong (+++) (Uysal et al., 2015).

### 2.10 Statistical analysis

SPSS version 24 was used for all statistical analysis. The data were expressed as mean ± SEM. Statistical significance was assessed by one‐way analysis of variance (ANOVA) and two way repeated measures ANOVA. Values of *p *<* *0.05 were considered statistically significant.

## 3 RESULTS

### 3.1 Open field test (OFT)

**Figure 1:**
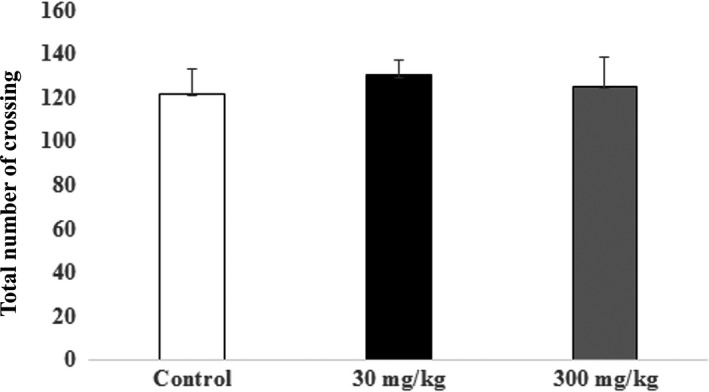
Open field test: Locomotor activity of rats was assessed in an open field test following 14 days of treatment with the extract of *Centella asiatica*. The graph shows the mean (±SEM) of the total number of crossing in the open field test. One‐way ANOVA showed no significant changes between control and treatments animals (*p *>* *0.05) with Cronbach's alpha: 0.999

The OFT was used to test the effect of selected doses of *C. asiatica* extract on the locomotor activity of the animals as motor deficit of animals may affect the escape latency in water T‐maze test. The OFT data showed that the mean of total number of crossings was 136.5 in the control group, 130.4 in the 30 mg/kg group, and 132 in the 300 mg/kg group. The numbers of crossings were similar and there was no significant difference across all study groups Therefore, the *C. asiatica* extract at doses of 30 and 300 mg/kg did not affect the rat locomotor or exploratory activity (Figure 1).

### 3.2 Water T‐maze (WTM)

#### 3.2.1 Learning acquisition phase

**Figure 2:**
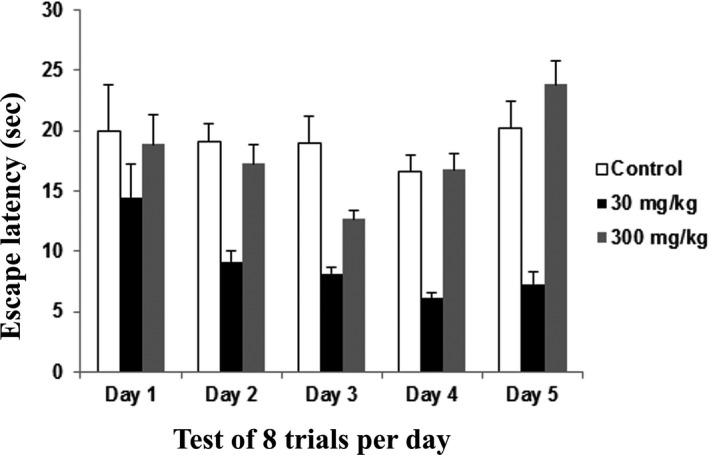
Water T maze – Learning acquisition phase of rats following 14 days of administration of the extract of *Centella asiatica* was assessed in water T‐maze. The graph shows the mean (±SEM) of escape latency of learning acquisition phase (sec) from day 1 to day 5. Two‐way repeated ANOVA showed significant (*) changes between control and treatments animals (*p *<* *0.05) with Cronbach's alpha: 0.977

The mean escape latency (s) for the learning acquisition phase of WTM across 5 days were as followed: control group [day 1 (19.94), day 2 (19.13), day 3 (19.02.), day 4 (16.60), and day 5 (20.25)], 30 mg/kg group [day 1 (14.46), day 2 (9.14), day 3 (8.13), day 4 (6.13), and day 5 (7.30)] and 300 mg/kg group [day 1 (18.91), day 2 (17.29), day 3 (12.68), day 4 (16.75), and day 5 (23.84)]. The control group revealed no differences and showed a fluctuating pattern of escape latency over 5 days. The 30 mg/kg group had a significant decrease in escape latency over the 5 day trial when compared to the control and 300 mg/kg dose group. However, the 300 mg/kg group showed a decreased latency until day 3 and an increased latency at day 4 and 5 of the trial. Thus, a dose of 30 mg/kg improved the learning acquisition phase in the water T‐maze test (Figure 2).

#### 3.2.2 Memory phase

**Figure 3:**
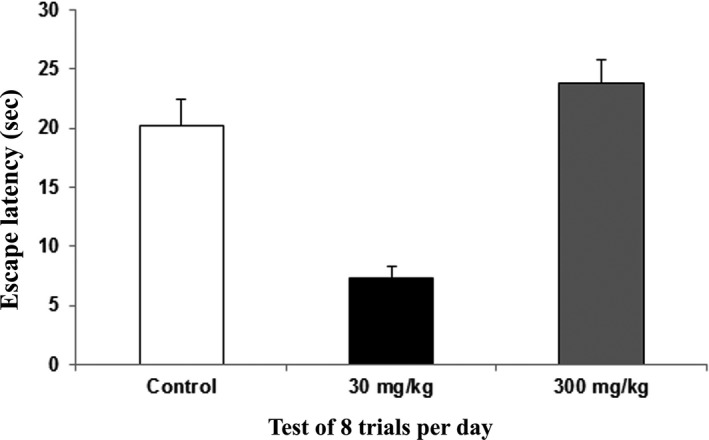
Water T‐maze – The memory phase of the rats following 14 days of administration of the extract of *Centella asiatica* was assessed with the water T‐maze. The graph shows the mean (±SEM) of escape latency of memory phase (sec). One way ANOVA showed significant (*) changes between the control and treatment animals (*p *<* *0.05) with Cronbach's alpha: 0.996

The memory phase was studied using data for the mean escape latency obtained on day 5. The control group value was 20.25 s, the 30 mg/kg group was 7.3 s, and the 300 mg/kg group was 23.84 s. The memory phase results showed that rats dosed at 30 mg/kg showed better learning and memory when compared to the rats in control group and the 300 mg/kg group. The memory test evaluates working memory, which is the ability to encode, maintain, and flexibly manipulate information that is no longer present in the environment. The working memory also includes information about abstract rules, recent events, and goals for future actions (Bizon, Foster, Alexander, & Glisky, 2012). The rats dosed at 30 mg/kg showed an improved memory phase and therefore had a better working memory when compared to the rats in the control and 300 mg/kg groups (Figure 3).

#### 3.2.3 Memory consolidation phase

**Figure 4:**
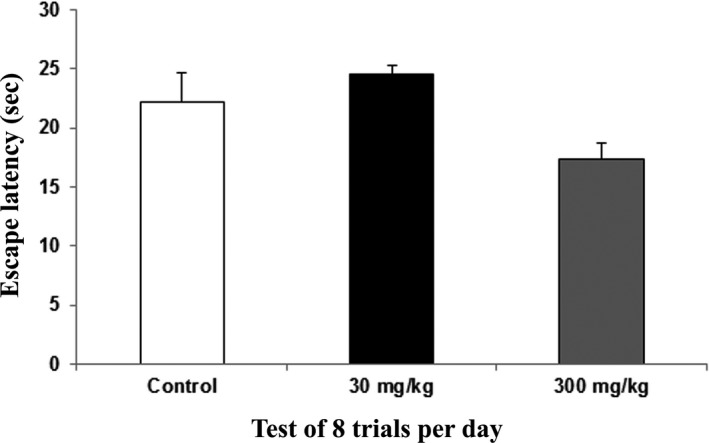
Water T‐maze – The memory consolidation phase of rats following 14 days of administration of the extract of *Centella asiatica* was assessed with the water T‐maze. The graph shows the mean (±SEM) of escape latency in the probe trial for memory consolidation. One way ANOVA showed significant (*) changes between the control and treatment animals (*p *<* *0.05) with Cronbach's alpha: 1.000

The probe trial was used to test for memory consolidation. The control group latency value was 22.25 s, the 30 mg/kg group value was 24.5 s, and the 300 mg/kg group value was 17.4 s. Memory consolidation is the neurological process whereby newly acquired information is gradually stabilized from an initial state of fluctuation to a permanent state, and it involves enhancement of the memory trace after the initial acquisition (Zhu et al., 2014). The results indicated that better memory consolidation was achieved in the 30 mg/kg group than in the control and 300 mg/kg groups (Figure 4).

#### 3.2.4 Reversal learning phase

**Figure 5:**
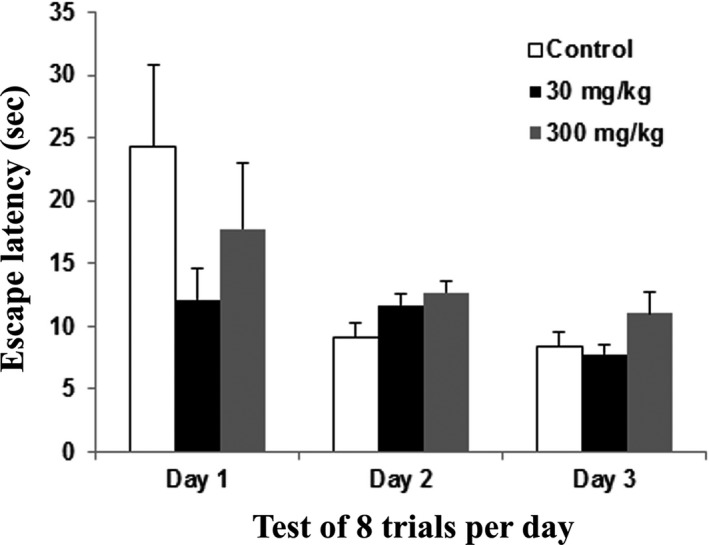
Water T‐maze – Reversal learning phase of rats following 14 days of administration of the extract of *Centella asiatica* was assessed with the water T‐maze. The graph shows the mean (±SEM) of escape latency in the reversal learning test. One way ANOVA showed no significant changes between the control and treatment animals (*p *>* *0.05) with Cronbach's alpha: 1.000

The reversal learning values for the mean of escape latency for 3 days were as followed: control group [day 1 (24.27), day 2 (9.08), and day 3 (8.31)], 30 mg/kg group [(day 1 (12.11), day 2 (11.65), and day 3 (7.79)], and 300 mg/kg group [(day 1 (17.78), day 2 (12.66), and day 3 (11.0)]. No significant changes were noted in each group, but the rats in the 30 mg/kg group had a more decreased latency pattern when compared to the rats in the control and 300 mg/kg groups (Figure 5).

### 3.3 Cresyl violet

**Figure 6:**
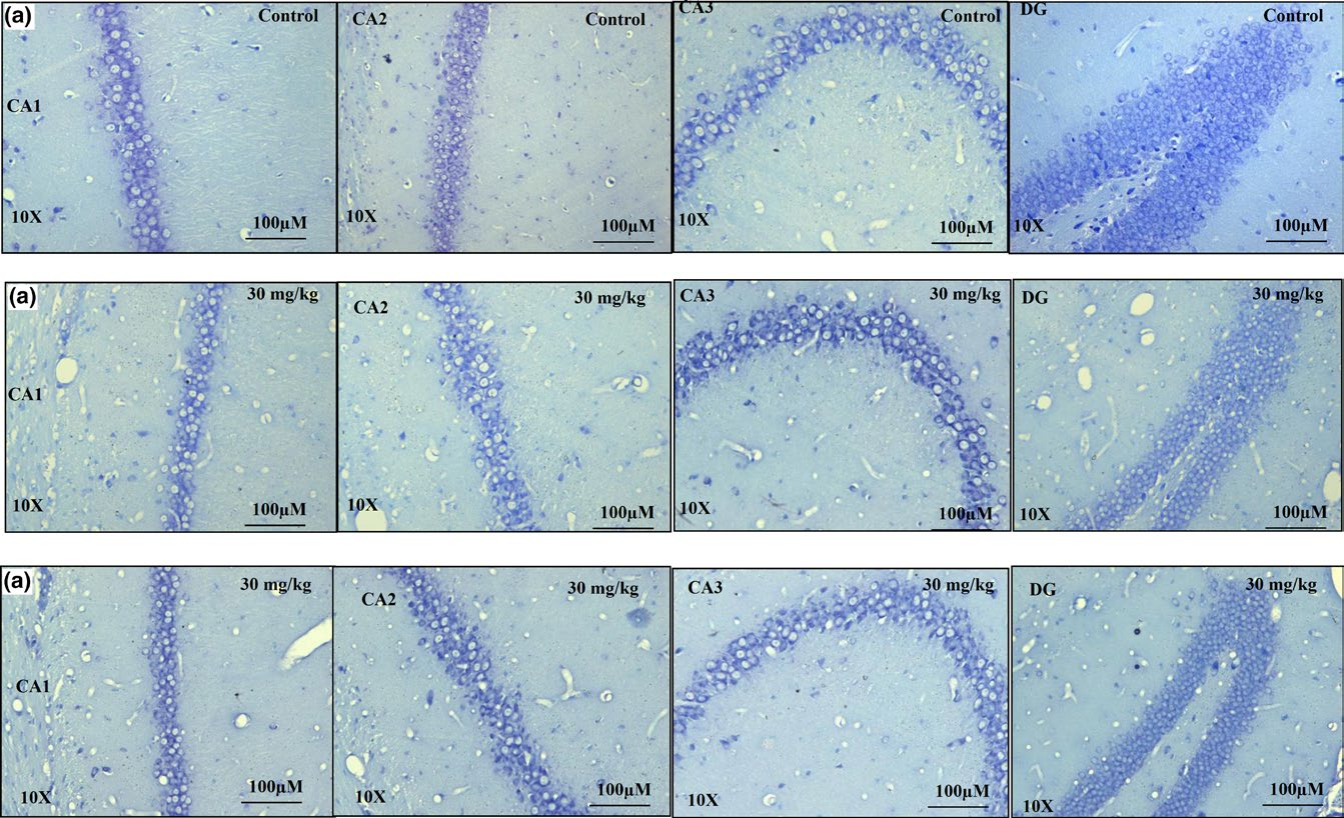
Cresyl violet stained sections of rat hippocampus subregions CA1, CA2, CA3, and DG from control (*n* = 5) (A), 30 mg/kg (*n* = 5) (B), and 300 mg/kg (*n* = 5) (C) groups following 14 days of administration of the extract of *Centella asiatica*. No significant changes in morphology were observed between control and treatment animals. All three blinded investigators agreed on their observations. Scale bar = 100 μm

Cresyl violet (CV) staining is a histological test for Nissl bodies and neuron nuclei and is used to evaluate morphological changes in the brain. Dead cells were identified morphologically by blebbing of the plasma membrane, diffuse pallor of the eosinophilic background, alterations in the size and shape of the cells, vacuolation, chromatin condensation, and nucleus condensation. The hippocampus in the control, 30 mg/kg, and 300 mg/kg groups showed cells with a normal appearance, including a well‐defined nuclear membrane with a clearly visible nucleolus, indicating that these were live cells (Moorthi et al., 2015) (Figure 6).

### 3.4 Apoptosis staining

**Figure 7:**
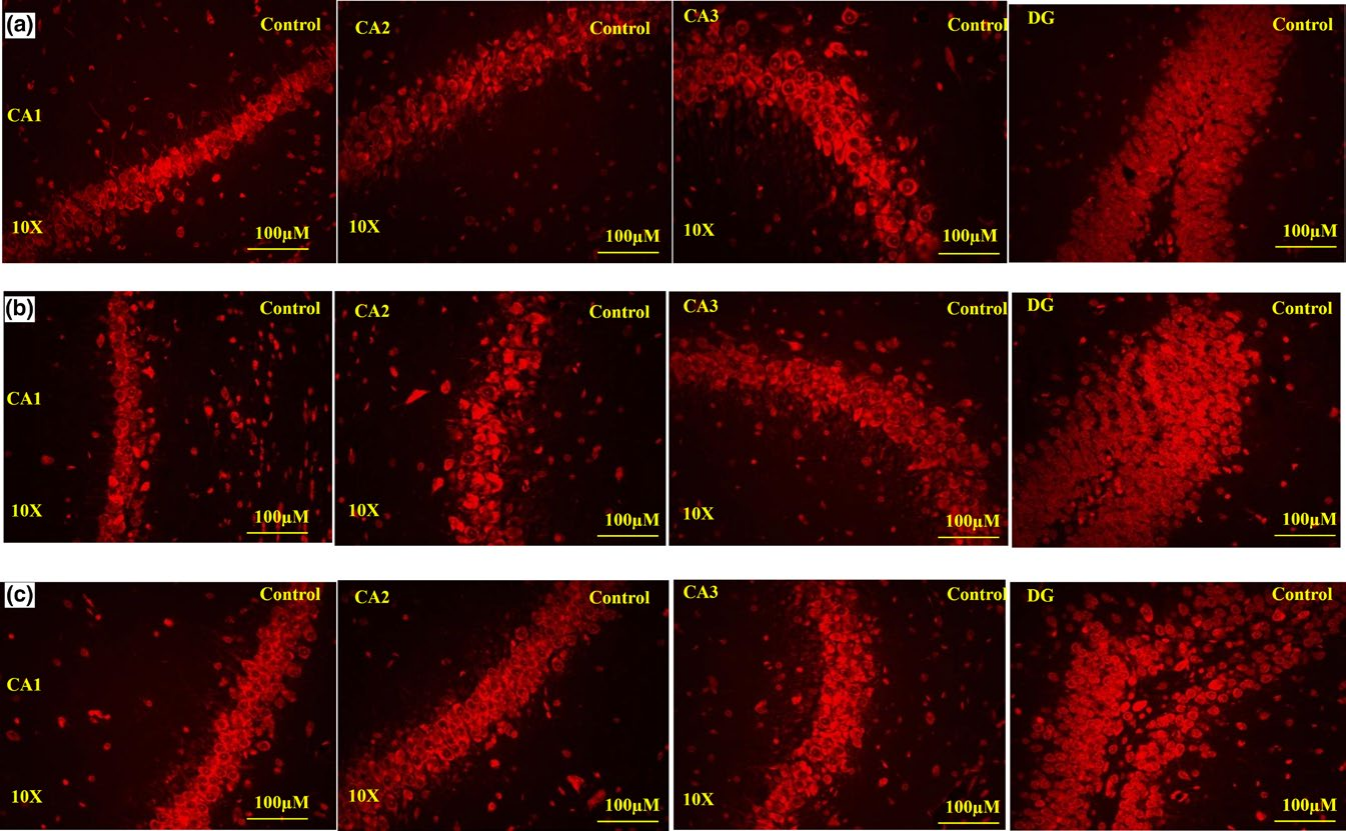
Apoptosis staining: Effect of administration of the extract of *Centella asiatica* (CA) for 14 days on the rat hippocampus sub regions CA1, CA2, CA3, and DG in control (*n* = 5) (A), 30 mg/kg (*n* = 5) (B), and 300 mg/kg (*n* = 5) (C) groups. No apoptosis occurred in the control or treatment animals. All three blinded investigators agreed on their observations. Scale bar = 100 μm

Apoptosis staining with Annexin V is typically used in conjunction with a vital dye, such as propidium iodide (PI), for identification of early and late apoptotic cells. Viable cells with intact membranes exclude PI, whereas the membranes of dead and damaged cells are permeable to PI. Therefore, cells that are considered viable are both Annexin V and PI negative, while cells that are in early apoptosis are Annexin V positive and PI negative, and cells that are in late apoptosis or already dead are both Annexin V and PI positive (Hingorani, Deng, Elia, McIntyre, & Mittar, 2011). In the present study, the hippocampus in the control, 30 mg/kg, and 300 mg/kg groups showed no evidence of apoptosis in any area of the hippocampus, as indicated by lack of staining with propidium iodide and Annexin V inside the plasma membrane (Figure 7).

### 3.5 HRP/DAB

**Figure 8:**
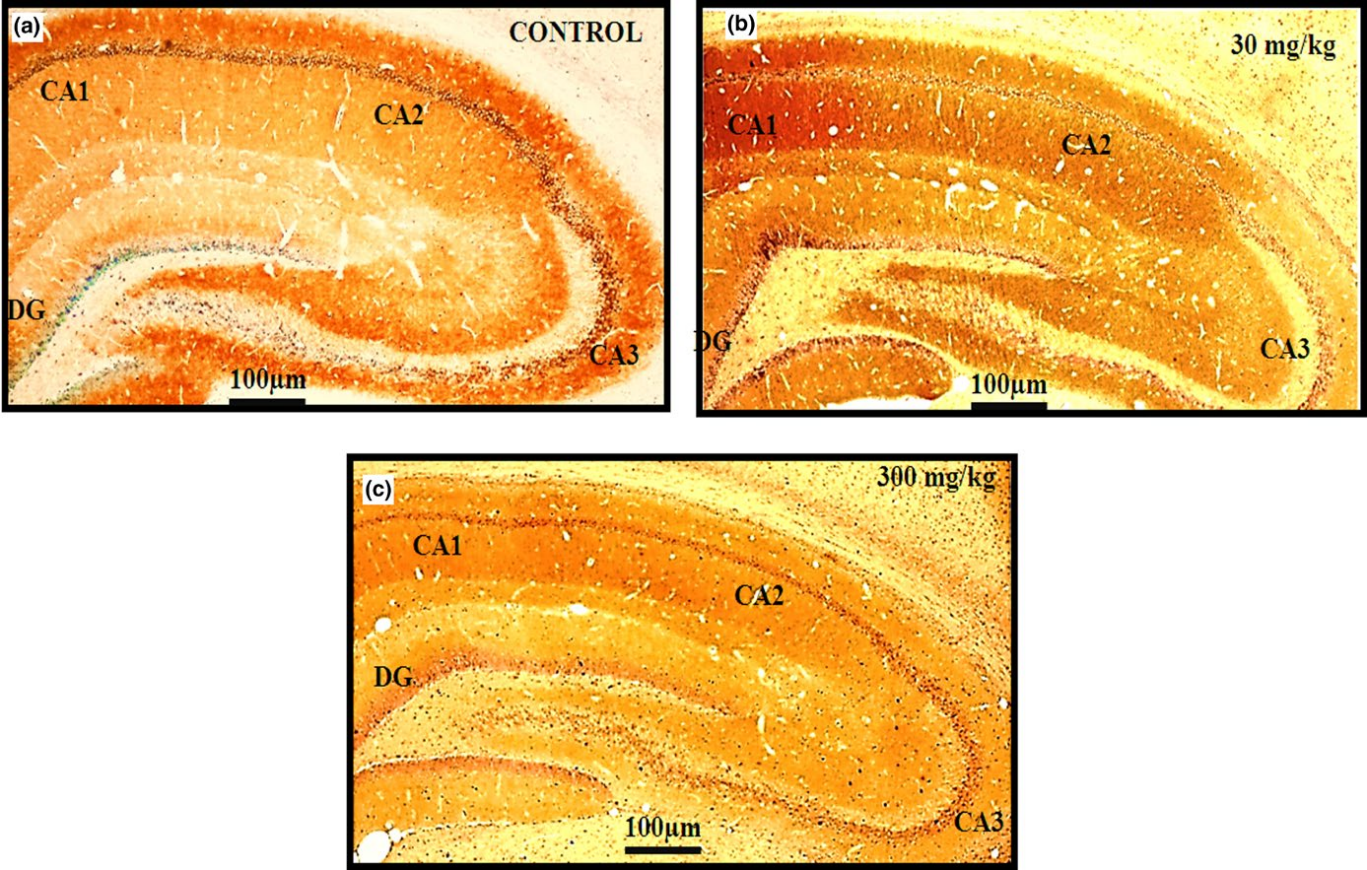
HRP/DAB staining – Immunohistochemical analysis for detection of the qualitative expression of the glutamate AMPA GluA1 receptor distribution in sections of rats hippocampus subregions CA1, CA2, CA3, and DG in control (*n* = 5) (A), 30 mg/kg (*n* = 5) (B), and 300 mg/kg (*n* = 5) (C) groups following 14 days of treatment with the extract of *Centella asiatica*. Image (A) shows moderate expression (++), (B) strong expression (+++) and (C) mild expression (+). The cells and tissues were labeled with the chromogen 3‐3‐diaminobenzidine (DAB). Three blinded investigators gave the same scale value in their observations. Cohen kappa: 1.000. Scale bar = 100 μm

**Figure 9:**
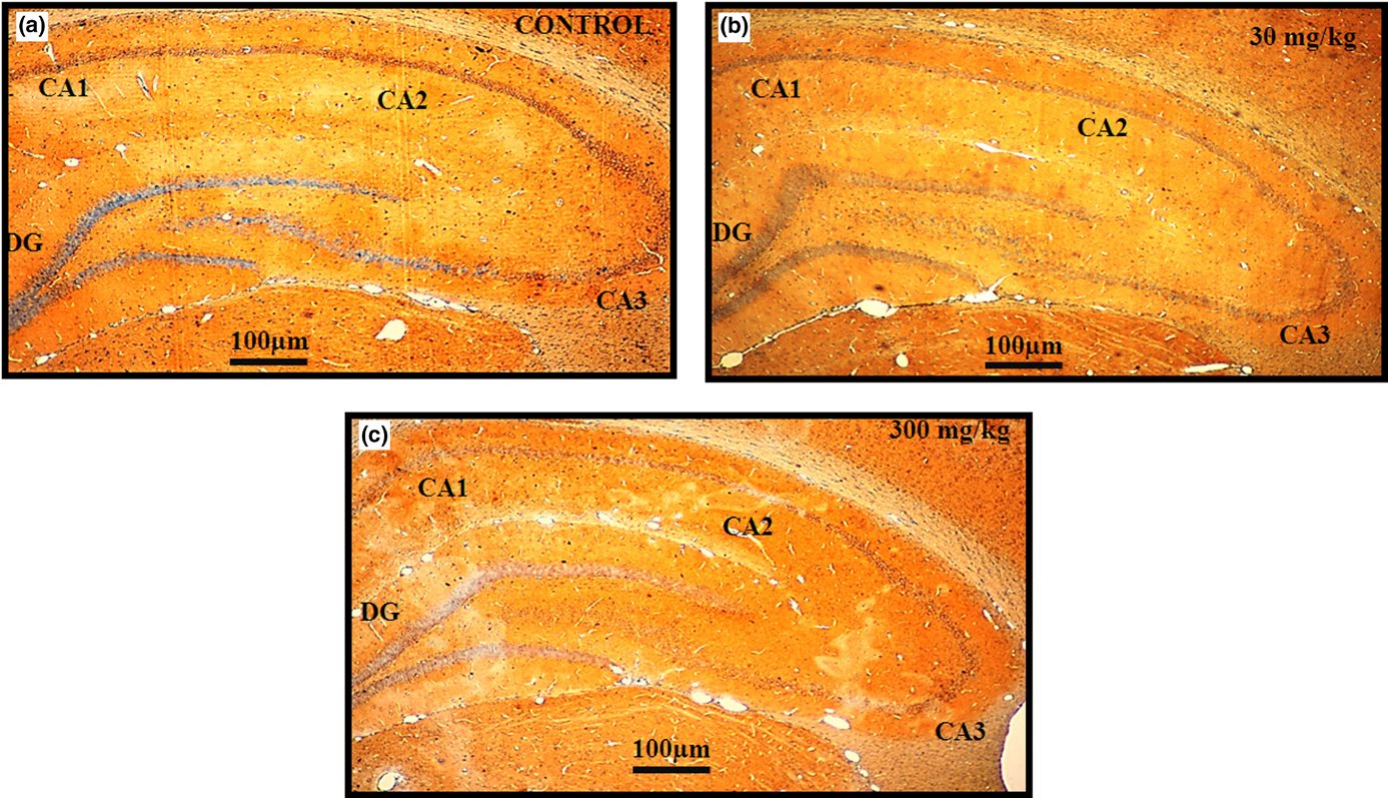
HRP/DAB staining – Immunohistochemical analysis for qualitative detection of the expression of the GABA_A_ α1 receptor subunit distribution in sections of rat hippocampus sub regions CA1, CA2, CA3, and DG in control (*n* = 5) (A), 30 mg/kg (*n* = 5) (B), and 300 mg/kg (*n* = 5) (C) groups of rats following 14 days of administration of the extract of *Centella asiatica*. Images (A, B, C) show moderate expression (++), with no significant difference between the control and treatment groups. The cells and tissues were labeled with the chromogen 3‐3‐diaminobenzidine (DAB). Three blinded investigators agreed on their observations. Scale bar = 100 μm

Immunohistochemical analysis was conducted to determine the qualitative distribution of the glutamate AMPA GluA1 receptor subunit in sections of the rat hippocampus sub regions CA1, CA2, CA3, and DG in the control (*n* = 5) (A), 30 mg/kg (*n* = 5) (B), and 300 mg/kg (*n* = 5) (C) groups following 14 days of treatment with *C. asiatica* extract. The images were graded as follows: (A) mild expression (+), (B) moderate expression (++), and (C) strong expression (+++).Three blinded investigators gave the same scale values for their observations (Cohen kappa: 1.000. Scale bar = 100 μm). The expression of AMPA GluA1 receptor in the rat hippocampus sections was greater in the 30 mg/kg group than in the control and 300 mg/kg groups, especially in the CA1 and CA2 sub regions (Figure 8). Immunohistochemical analysis was also conducted for qualitative determination of the distribution of GABA_A_ α1 receptor subunit expression in sections of the hippocampus sub regions CA1, CA2, CA3, and DG in the control (A), 30 mg/kg (B), and 300 mg/kg (C) groups of rats following 14 days of treatment with the *C. asiatica* extract. Images for A, B, and C show moderate expression (++), with no significant changes between the control and treatment groups (Scale bar = 100 μm). Three blinded investigators agreed in their observations. The expression of the GABA_A_ α1 receptor subunit in the rat hippocampus sections did not differ among the three groups (Figure 9).

## 4 DISCUSSION

Many herbal plants are reported to improve learning and memory. *Centella asiatica* has many beneficial effects: in fact, the most widely reported health benefit of this herb is its improvement of brain function, particularly related to memory and learning, as the whole plant is very useful in improving these functions (Sari et al., 2014; Sirichoat et al., 2015; Yolanda et al., 2015). These benefits are due to the protective effect of the phytochemicals contained in the *C. asiatica* plant. Extracts from *C. asiatica* provide antioxidant properties due to the content of triterpenoids, such as asiaticoside, madecassoside, asiatic acid, and madecassic acid, and phenolics compounds, as reported previously (Nasir et al., 2015; Orhan, 2012; Sari et al., 2014; Tripathi, Mishra, Upadhyay, Purohit, & Dubey, 2015). Yolanda et al. (2015) stated that some of these active substances of *C. asiatica,* including asiatic acid, madecassic acid, asiaticoside, and madecassoside, may be able to penetrate the blood brain barrier. In addition, this plant contains phenolic constituents that are powerful antioxidants believed to have protective activity (Abas, Khatib, Shaari, & Lajis, 2014; Thoo et al., 2013; Tripathi et al., 2015). The present results indicated an improvement in learning and memory in adolescent rats, as shown by the results of the behaviour tests that specified the learning acquisition, memory phase, and memory consolidation phase, whereas locomotor activity and reversal learning were not affected by the administration of *C. asiatica*.

Adolescence is a period of human psychological and physical development. It is also a period of gradual transitioning of behavioral and cognitive functions from childhood to adulthood, as adult behavioral abilities are acquired between the onset of puberty and adulthood (Mengler et al., 2014; Schneider, 2013). During adolescence, drastic changes in neuronal architecture and function occur that concomitantly lead to distinct behavioral alteration. This alteration makes the adolescent development period a time of vulnerability and adjustment in psychological and physiological areas (Arain et al., 2013; Mengler et al., 2014; Schneider, 2013). Changes in nutrition may also affect neuronal structure and function, as well as neuron connectivity, leading to a long‐lasting beneficial effect (Beheshti et al., 2016).

The open field test results showed that the *C. asiatica* extract does not cause significant differences in locomotor activity. Similarly, an earlier study (Gupta, Kumar, & Srivastava, 2003) confirmed that locomotor activity in rats induced with *C. asiatica* at doses of 100 and 300 mg/kg did not differ significantly. In another study, Nasir et al. (2015) showed that administration of the components of *C. asiatica –* specifically asiatic acid, madecassic acid, and madecassoside – in adolescents resulted in improved spatial memory with no sedative effects on locomotor activity. Similarly, the study by Sharma and Gupta (2016) showed that treatment of mice with doses of *C. asiatica* extract of 50, 100, 200, and 500 mg/kg resulted in no significant changes in locomotor activity (*p *>* *0.05). Therefore, these effects were not attributable to changes in motor function or in the autonomic nervous system, as shown in both mice and rats.

Histological sections of the rat hippocampus stained with cresyl violet showed no differences in morphology and no damage in all groups. The sections stained to reveal apoptosis showed clear neuronal nuclei by microscopy with a 20 ×  power lens. No apoptosis or cell death occurred in the rats in all groups, and the cell nuclei remained intact. Thus, our *C. asiatica* extract apparently caused no damage to the hippocampal area of the rat brain.

The therapeutic dose range of *C. asiatica* is wide. Chivapat, Chavalittumrong, and Tantisira (2011) showed in their studies of acute and subchronic toxicity studies of a standardized extract of *C. asiatica* that a dose of up to 10.0 g/kg produced no acute toxicity, while no significant subchronic toxicity was observed in rats receiving 10 to 1,000 mg/kg. Abdulla, Al‐Bayaty, Younis, and Hassan (2010) showed that doses of 2 and 5 g/kg of *C. asiatica* leaf extracts did not manifest any significant visible signs of toxicity in rats, and they concluded that the extract is safe even at these high doses and that the oral lethal dose (LD50) for male and female rats was greater than 5 g/kg body weight. The acute toxicity study conducted by Rahman, Sayeed, Haque, Hassan, and Islam (2012) confirmed that administration of *C. asiatica* to rats, even at a dose of 300 mg/kg, was safe and well‐tolerated, as no deaths or toxicity signs occurred. Further support comes from the results of the toxicity study by Deshpande, Mohan, and Thakurdesai (2015), who conducted a preclinical safety assessment of a standardized extract of *C. asiatica*. They showed that a dose of this standardized extract had no significant toxic effects and was found safe up to a dose of 2,000 mg/kg.

The water T‐maze test was administered to test the learning and memory function of the rats, specifically because the working memory involves mainly the hippocampus. The ability of an individual to remember spatial locations in the short‐term depends on spatial working memory. This is a critical cognitive function and can be assessed using various maze‐based tasks, including the T‐maze (O'Neill, Gordon, & Sigurdsson, 2013; Spellman et al., 2015). The water T‐maze, developed by Del Arco, Segovia, Garrido, de Blas, and Mora (2007), involves an animal's choice of an arm consisting of a platform that allows an escape from water. The water T‐maze works from the same principle as Morris water maze where it is the animal's innate nature to search for escape from waters. The main advantage of this technique is that it is free from food or water deprivation and promotes a simple motivating stimulus by escaping from water. This technique also may reduce the duration of the test (Del Arco et al., 2007; Locchi et al., 2007; Vorhees & Williams, 2014). Our results showed learning and memory improvement in the rats dosed at 30 mg/kg in terms of the learning acquisition, memory, and memory consolidation phases. However, no significant difference was noted in reversal learning. The previous study also supported our present results that *C. asiatica* improved memory of rats but did not enhance learning, (Jared, 2010) while in contrast, our results revealed that learning was also improved following *C. asiatica* treatment.

The enhancement of learning and memory by *C. asiatica* extract was also reported by Rao, Chetana, and Devi (2005), who found that administration of 200 mg/kg dose of *C. asiatica* extract during the postnatal period resulted in increased brain function in juvenile and young adult mice. Another study by Prakash and Kumar (2013) showed that administration of *C. asiatica* at doses of 150 and 300 mg/kg significantly improved memory performance. The learning and memory effect of *C. asiatica* was also evident in the study by Rao, Rao, and Rao (2012), who showed that administration of *C. asiatica* extract to adult rats facilitated dendritic growth and increased the dendritic length and branches of the neurons of the amygdala. Therefore, the improvement in learning and memory of the treated rats was due to the enhancement of dendritic arborization. However, the increase in dendritic intersections and branches of the amygdaloid neurons was only seen in animals treated with *C. asiatica* at doses of 6 ml/kg consecutively for 6 weeks. This result suggests that higher amounts of *C. asiatica* extract are needed to induce structural changes in the neurons; therefore, lower doses of *C. asiatica* extract and shorter durations of treatment failed to induce structural changes. These findings supported our results, as we found no changes in morphology (Rao et al., 2012) or in reversal learning.

Reversal learning refers to the cognitive flexibility that is required to reverse the learning of previously acquired behavioral acquisition learning. It is the ability to adjust behavior to changes in the environment or task conditions, and it is measured as the capacity for reversal learning of an acquired task. Cognitive flexibility is the capacity and ability of humans to adapt their cognitive processing strategies to face a new and unexpected condition in the environment and to undergo new stimulus adaptation that involves modification of working memory, attention, and response selection as a result of changing endogenous and exogenous task demands (Bizon et al., 2012; Deak & Wiseheart, 2015). Thus, if rats were able to switch or shift their thinking from one dimension to another dimension quickly, this indicated a greater level of cognitive flexibility. This suggests that the rats in all groups showed better cognitive flexibility and no significant difference existed between the control and the treatment groups. Leuner and Gould (2010) stated that the improved cognitive functions specifically during postpartum period can be associated with the medial prefrontal cortex (mPFC), suggesting that spine density changes may underlie improved cognitive ability that triggered by synaptic activity. They found enhanced dendritic spine density and improved cognitive flexibility during the postpartum period. In relation to this statement, the study of Rao et al. (2012) indicated that only high doses of *C. asiatica* extract induced structural changes, including dendritic spine changes. These findings supported our result in which selected doses of standardized crude extract of *Centella asiatica* were unable to induce dendritic arborization, which in turn led to a lack of a difference or significant improvement in reversal learning between the control and treated groups.

Animal studies by Blazevic, Colic, Culig, and Hranilovic (2012) also indicated that serotonin (5HT) plays a major role in the modulation of reversal learning, as depletion of serotonin can impair cognitive flexibility. We found no impairment of cognitive flexibility; thus, the *C. asiatica* extract does not decrease the level of serotonin. However, the lack of significant improvement in reversal learning may indicate that the amount of *C. asiatica* extract administered was insufficient to alter serotonin activities. Another findings of *C. asiatica* extract administration as a chronic treatment was that the extract reversed D‐galactose induced AChE activity in aging mice, indicating that this extract may improve dysfunction of the cholinergic system due to long‐term exposure to oxidative stress (Kumar, Prakash, & Dogra, 2011). Nasir, Abdullah, Habsah, Ghani, and Rammes (2012) showed that asiatic acid isolated from *C. asiatica* was a cognitive enhancer and had an inhibitory effect on acetylcholinesterase and selective GABA_B_ receptor agonist activity in rats. These findings were also supported by Kumar, Dogra, and Prakash (2009), who found that administration of *C. asiatica* extract at doses of 150 and 300 mg/kg ameliorated the colchicine effect by decreasing the AChE activity in rats.

The hippocampus is a regulator of spatial learning and memory. Glutamate is a key excitatory neurotransmitter in the hippocampus and plays a central role in the activation of the trisynaptic circuit. By contrast, the inhibitory neurotransmitter GABA modulates the activated circuit. The balance between the excitatory and inhibitory actions is critical for the appropriate functioning of the hippocampal circuit (Iwata & Yamamuro, 2016). The hippocampus consists of three topographically and morphologically distinct neuronal layers: the pyramidal cell layer in subfields CA1 and CA3 and the granule cell layer in the dentate gyrus (DG) (Iwata & Yamamuro, 2016). Thus, in our study, an essential step was to evaluate the expression of AMPA GluA1 and GABA_A_ α1 receptor subunits in the hippocampus. Our immunohistochemistry result showed that AMPA GluA1 receptors were strongly expressed in the CA1 and CA2 areas in the 30 mg/kg group, when compared to the control and 300 mg/kg groups.

The AMPA receptors (AMPARs) are a subtype of the ionotropic glutamate receptors. Glutamate is the main and most abundant excitatory neurotransmitter in the CNS and has key roles in several physiological functions. Most AMPAR are heterotetrameric, consisting of combinations of GluA1, GluA2, GluA3, and GluA4 subunits. Approximately 80% of synaptic AMPARs in the CA1 hippocampal neurons consist of GluA1 and GluA2 heteromers. The GluA1 subunit is highly expressed in the hippocampus, central amygdala, and cerebellum (Anggono & Huganir, 2012; Henley & Wilkinson, 2016; Inta et al., 2014; Sanderson & Bannerman, 2012). These AMPARs are critically important for nearly all aspects of brain function, including learning, memory, and cognition, as they mediate the overwhelming majority of fast excitatory neurotransmission in the CNS (Henley & Wilkinson, 2013).

GluA1 is particularly important for structural and functional plasticity, as it induces structurally stabilizing effects and increases synaptic strength. GluA1 is the main molecular determinant of LTP and synaptic plasticity and, therefore, of learning and memory (Inta et al., 2014). This may explain why AMPA GluA1 receptor expression in hippocampus was increased by 30 mg/kg dose but not by 300 mg/kg dose. This idea is supported by the results for rats dosed at 30 mg/kg, as these rats showed increases in a spatial learning task in the learning acquisition phase, the memory phase, and the memory consolidation phase. By contrast, dosing at 300 mg/kg did not elicit a similar improvement in AMPA GluA1 receptor expression, perhaps because of receptor desensitization, in which a decreased receptor response to signaling molecules occurs when the agonist is present at high concentrations. According to Robert and Howe (2003), AMPARs can desensitize within a few milliseconds in the sustained presence of glutamate. Glutamate‐induced desensitizations are more sensitive to GluA1 channels and their desensitizations recover more slowly, primarily because GluA1 channels enter desensitization more readily and resensitize more slowly. This could explain the lower expression of AMPA GluA1 in rats given the 300 mg/kg dose, as a higher concentration of glutamate produced by the extract caused desensitization of the receptor in the adolescent age. GABA is implicated in many processes of neurogenesis, including neuronal proliferation, migration, differentiation, and preliminary circuit‐building, as well as the development during critical periods (Wu & Sun, 2015). In the mature CNS of the adult brain, GABA acts as an inhibitory neurotransmitter via activation of the fast hyperpolarizing GABA_A_ receptors. When GABA binds to these receptors at the postsynaptic site, an ion channel opens and chloride ions (Cl^−^) diffuses into the cell along its concentration gradient, thus hyperpolarizing the postsynaptic mature neuron (Wu & Sun, 2015). The major phytochemical compounds, such as asiatic acid found in *C. asiatica* has an inhibitory effect on acetylcholinesterase and selective GABA_B_ receptor agonist activity (Ceremuga et al., 2015). Asiatic acid acts as AChE inhibitor to improve the levels of ACh.

Our current study showed that *Centella asiatica* improves learning and memory by modulating AMPA but not GABA in hippocampus. Another study by our department revealed that there was difference between AMPARs and GABARs involvement in the mediation of memory performance. The results from electrophysiological studies using whole‐cell patch clamp technique on pyramidal neurons of the entorhinal cortex demonstrated that acute application of *Centella asiatica* extract significantly increased the amplitude of the glutamatergic spontaneous excitatory postsynaptic currents (sEPSCs) mediated by AMPARs while this effect was not observed in GABAergic currents. Further study in our lab through immunohistochemical analysis also showed that there was an increase of AMPARs subunits, targeting the GluA1 and GluA2 subunits which are crucial for synaptic plasticity and play an important role in the modulation of learning and memory behavior. The increased expression of AMPARs leads to increase in conductance which is reflected in the higher sEPSCs amplitude between control and treated cells (Unpublished data).

ACh has an excitatory role in the hippocampus area due to its involvement in excitation of hippocampal pyramidal neurons. Pharmacological activation of hippocampal muscarinic ACh receptors also directly excites GABAergic interneurons (Nasir et al., 2015). By contrast, Shen et al. (2017) reported that, at the onset of puberty, expression of α4βδ GABA_A_ receptors (GABA_A_Rs) increases on dendritic spines of CA1 hippocampal pyramidal cells. These receptors reduce activation of NMDA receptors (NMDARs), impair induction of LTP, and reduce hippocampal‐dependent spatial learning. Activation of GABA receptors impairs learning and memory, and this could explain why the *C. asiatica* extract increases the levels of excitatory neurotransmitters, such as acetylcholine, that aid in improving learning and memory, but at higher doses it may cause the excitation of GABAergic interneurons without affecting the expression of GABA_A_ receptors. This could possibly explain why 300 mg/kg dose of *C. asiatica* extract does not provide the same learning and memory improvements observed with 30 mg/kg dose.

## 5 CONCLUSIONS

In conclusion, administration of the *C. asiatica* extract improved hippocampus‐dependent spatial learning and memory in a dose‐dependent manner in rats through increased expression of the AMPA GluA1 receptor in the CA1 and CA2 regions of the hippocampus. Further studies on this extract and others receptors and neurotransmitters are recommended in order to determine effects on cognitive functions, specifically in learning and memory. Intensive research is needed to identify the underlying molecular mechanism by which the *C. asiatica* extract prevents pathological alterations and protects the brain from neurodegeneration and, more importantly, how it improves cognitive function.

## CONFLICT OF INTEREST

No author has conflict of interest.

## References

[brb31093-bib-0001] Abas, F. , Khatib, A. , Shaari, K. , & Lajis, N. H. (2014). Chemical characterization and antioxidant activity of three medicinal Apiaceae species. Industrial Crops and Products, 55, 238–247.

[brb31093-bib-0002] Abdulla, M. A. , Al‐Bayaty, F. H. , Younis, L. T. , & Hassan, M. A. (2010). Anti‐ulcer activity of *Centella asiatica* leaf extract against ethanol‐induced gastric mucosal injury in rats. Journal of Medicinal Plants Research, 4(13), 1253–1259.

[brb31093-bib-0004] Al‐Rahbi, B. , Zakaria, R. , Othman, Z. , Hassan, A. , Ismail, Z. I. M. , & Muthuraju, S. (2014). Tualang honey supplement improves memory performance and hippocampal morphology in stressed ovariectomized rats. Acta Histochemica, 116(1), 79–88. 10.1016/j.acthis.2013.05.004 23810156

[brb31093-bib-0005] Anggono, V. , & Huganir, R. L. (2012). Regulation of AMPA receptor trafficking and synaptic plasticity. Current Opinion in Neurobiology, 22(3), 461–469. 10.1016/j.conb.2011.12.006 22217700 PMC3392447

[brb31093-bib-0006] Arain, M. , Haque, M. , Johal, L. , Mathur, P. , Nel, W. , Rais, A. , … Sharma, S. (2013). Maturation of the adolescent brain. Neuropsychiatric Disease and Treatment, 9, 449–461. 23579318 10.2147/NDT.S39776 PMC3621648

[brb31093-bib-0007] Beheshti, F. , Hosseini, M. , Vafaee, F. , Shafei, M. N. , & Soukhtanloo, M. (2016). Feeding of *Nigella sativa* during neonatal and juvenile growth improves learning and memory of rats. Journal of Traditional and Complementary Medicine, 6(2), 146–152. 10.1016/j.jtcme.2014.11.039 27114937 PMC4833462

[brb31093-bib-0008] Bizon, J. L. , Foster, T. C. , Alexander, G. E. , & Glisky, E. L. (2012). Characterizing cognitive aging of working memory and executive function in animal models. Frontiers in Aging Neuroscience, 4, 19. 22988438 10.3389/fnagi.2012.00019 PMC3439637

[brb31093-bib-0009] Blazevic, S. , Colic, L. , Culig, L. , & Hranilovic, D. (2012). Anxiety‐like behavior and cognitive flexibility in adult rats perinatally exposed to increased serotonin concentrations. Behavioural Brain Research, 230(1), 175–181. 10.1016/j.bbr.2012.02.001 22342491

[brb31093-bib-0010] Ceremuga, T. E. , Valdivieso, D. , Kenner, C. , Lucia, A. , Lathrop, K. , Stailey, O. , … Taylor, A. (2015). Evaluation of the anxiolytic and antidepressant effects of asiatic acid, a compound from Gotu kola or *Centella asiatica*, in the male Sprague Dawley rat. AANA Journal, 83(2), 91–98. 26016167

[brb31093-bib-0011] Chakravarthi, K. K. , & Avadhani, R. (2013). Beneficial effect of aqueous root extract of Glycyrrhiza glabra on learning and memory using different behavioral models: An experimental study. Journal of Natural Science, Biology and Medicine, 4(2), 420 10.4103/0976-9668.117025 PMC3783792 24082744

[brb31093-bib-0012] Cherubini, E. , & Miles, R. (2015). The CA3 region of the hippocampus: How is it? What is it for? How does it do it? Frontiers in Cellular Neuroscience, 9, 1–3. 10.3389/fncel.2015.00019 25698930 PMC4318343

[brb31093-bib-0013] Chivapat, S. , Chavalittumrong, P. , & Tantisira, M. H. (2011). Acute and sub‐chronic toxicity studies of a standardized extract of *Centella asiatica* ECa 233. Thai Journal of Pharmaceutical Sciences, 35, 55–64.

[brb31093-bib-0014] Danysz, W. , & Parsons, C. G. (2012). Alzheimer's disease, β‐amyloid, glutamate, NMDA receptors and memantine–searching for the connections. British Journal of Pharmacology, 167(2), 324–352. 10.1111/j.1476-5381.2012.02057.x 22646481 PMC3481041

[brb31093-bib-0015] Deak, G. O. , & Wiseheart, M. (2015). Cognitive flexibility in young children: General or taskspecific capacity? Journal of Experimental Child Psychology, 138, 31–53. 10.1016/j.jecp.2015.04.003 26026421

[brb31093-bib-0016] Del Arco, A. , Segovia, G. , Garrido, P. , de Blas, M. , & Mora, F. (2007). Stress, prefrontal cortex and environmental enrichment: Studies on dopamine and acetylcholine release and working memory performance in rats. Behavioural Brain Research, 176(2), 267–273. 10.1016/j.bbr.2006.10.006 17097747

[brb31093-bib-0017] Deshpande, P. O. , Mohan, V. , & Thakurdesai, P. (2015). Preclinical safety assessment of standardized extract of *Centella asiatica* (L.) urban leaves. Toxicology International, 22(1), 10. 26862255 10.4103/0971-6580.172251 PMC4721154

[brb31093-bib-0018] Doknark, S. , Mingmalairak, S. , Vattanajun, A. , Tantisira, B. , & Tantisira, M. H. (2014). Study of ameliorating effects of ethanolic extract of *Centella asiatica* on learning and memory deficit in animal models.. Journal of the Medical Association of Thailand, 97, S68–S76. 25518178

[brb31093-bib-0020] Fields, R. D. , Araque, A. , Johansen‐Berg, H. , Lim, S. S. , Lynch, G. , Nave, K. A. , & Wake, H. (2014). Glial biology in learning and cognition. The Neuroscientist, 20(5), 426–431. 10.1177/1073858413504465 24122821 PMC4161624

[brb31093-bib-0021] Giribabu, N. , Srinivasarao, N. , Swapna Rekha, S. , Muniandy, S. , & Salleh, N. (2014). *Centella asiatica* attenuates diabetes induced hippocampal changes in experimental diabetic rats. Evidence‐Based Complementary and Alternative Medicine, 2014, 592062. 25161691 10.1155/2014/592062 PMC4139016

[brb31093-bib-0022] Gray, N. E. , Harris, C. J. , Quinn, J. F. , & Soumyanath, A. (2016). *Centella asiatica* modulates antioxidant and mitochondrial pathways and improves cognitive function in mice. Journal of Ethnopharmacology, 180, 78–86. 10.1016/j.jep.2016.01.013 26785167 PMC4764102

[brb31093-bib-0023] Gray, N. E. , Zweig, J. A. , Murchison, C. , Caruso, M. , Matthews, D. G. , Kawamoto, C. , & Soumyanath, A. (2017). *Centella asiatica* attenuates Aβ‐induced neurodegenerative spine loss and dendritic simplification. Neuroscience Letters, 646, 24–29. 10.1016/j.neulet.2017.02.072 28279707 PMC5533098

[brb31093-bib-0024] Guariglia, S. R. , & Chadman, K. K. (2013). Water T‐maze: A useful assay for determination of repetitive behaviors in mice. Journal of Neuroscience Methods, 220(1), 24–29. 10.1016/j.jneumeth.2013.08.019 23994357

[brb31093-bib-0025] Gupta, Y. K. , Kumar, M. V. , & Srivastava, A. K. (2003). Effect of *Centella asiatica* on pentylenetetrazole‐induced kindling, cognition and oxidative stress in rats. Pharmacology Biochemistry and Behavior, 74(3), 579–585. 10.1016/S0091-3057(02)01044-4 12543222

[brb31093-bib-0026] Hammerslag, L. R. , & Gulley, J. M. (2014). Age and sex differences in reward behavior in adolescent and adult rats. Developmental Psychobiology, 56(4), 611–621. 10.1002/dev.21127 23754712 PMC4782597

[brb31093-bib-0028] Henley, J. M. , & Wilkinson, K. A. (2013). AMPA receptor trafficking and the mechanisms underlying synaptic plasticity and cognitive aging. Dialogues in Clinical Neuroscience, 15(1), 11–27. 23576886 10.31887/DCNS.2013.15.1/jhenley PMC3622464

[brb31093-bib-0029] Henley, J. M. , & Wilkinson, K. A. (2016). Synaptic AMPA receptor composition in development, plasticity and disease. Nature Reviews Neuroscience, 17(6), 337–350. 10.1038/nrn.2016.37 27080385

[brb31093-bib-0030] Hingorani, R. , Deng, J. , Elia, J. , McIntyre, C. , & Mittar, D. (2011). Detection of apoptosis using the BD Annexin V FITC assay on the BD FACSVerse^™^ system. BD Biosciences, 1, 1–12.

[brb31093-bib-0031] Hsu, T. M. , Konanur, V. R. , Taing, L. , Usui, R. , Kayser, B. D. , Goran, M. I. , & Kanoski, S. E. (2015). Effects of sucrose and high fructose corn syrup consumption on spatial memory function and hippocampal neuroinflammation in adolescent rats. Hippocampus, 25(2), 227–239. 10.1002/hipo.22368 25242636

[brb31093-bib-0032] Inta, D. , Vogt, M. A. , Elkin, H. , Weber, T. , Lima‐Ojeda, J. M. , Schneider, M. , … Gass, P. (2014). Phenotype of mice with inducible ablation of GluA1 AMPA receptors during late adolescence: Relevance for mental disorders. Hippocampus, 24(4), 424–435. 10.1002/hipo.22236 24339333

[brb31093-bib-0034] Iwata, H. , & Yamamuro, Y. (2016). Subregional expression of hippocampal glutamatergic and GABAergic genes in F344 rats with social isolation after weaning. Comparative Medicine, 66(1), 4–9. 26884404 PMC4752030

[brb31093-bib-0035] Jared, S. R. (2010). Enhancement of memory in rats with Centella asiatica. Biomedical Research, 21(4), 429–432.

[brb31093-bib-0037] Krebs, C. , Weinberg, J. , & Akesson, A. (2012). Neuroscience (pp. 1–434). Maryland, USA: Lippincott Williams & Wilkins.

[brb31093-bib-0038] Kumar, A. , Dogra, S. , & Prakash, A. (2009). Neuroprotective effects of Centella asiatica against intracerebroventricular colchicine‐induced cognitive impairment and oxidative stress. International Journal of Alzheimer's Disease, 2009, 1–8. 10.4061/2009/972178 PMC2925281 20798885

[brb31093-bib-0039] Kumar, A. , Prakash, A. , & Dogra, S. (2011). *Centella asiatica* attenuates D‐galactose‐induced cognitive impairment, oxidative and mitochondrial dysfunction in mice. International Journal of Alzheimer's Disease, 2011, 1–9. 10.4061/2011/347569 PMC3100561 21629743

[brb31093-bib-0040] Leuner, B. , & Gould, E. (2010). Dendritic growth in medial prefrontal cortex and cognitive flexibility are enhanced during the postpartum period. Journal of Neuroscience, 30(40), 13499–13503. 10.1523/JNEUROSCI.3388-10.2010 20926675 PMC2963448

[brb31093-bib-0041] Li, M. , Masugi‐Tokita, M. , Takanami, K. , Yamada, S. , & Kawata, M. (2012). Testosterone has sublayer‐specific effects on dendritic spine maturation mediated by BDNF and PSD‐95 in pyramidal neurons in the hippocampus CA1 area. Brain Research, 1484, 76–84. 23010313 10.1016/j.brainres.2012.09.028

[brb31093-bib-0042] Locchi, F. , Dall'Olio, R. , Gandolfi, O. , & Rimondini, R. (2007). Water T‐maze, an improved method to assess spatial working memory in rats: Pharmacological validation. Neuroscience Letters, 422(3), 213–216. 10.1016/j.neulet.2007.06.023 17629404

[brb31093-bib-0043] Mengler, L. , Khmelinskii, A. , Diedenhofen, M. , Po, C. , Staring, M. , Lelieveldt, B. P. , & Hoehn, M. (2014). Brain maturation of the adolescent rat cortex and striatum: Changes in volume and myelination. NeuroImage, 84, 35–44. 10.1016/j.neuroimage.2013.08.034 23994458

[brb31093-bib-0044] Moorthi, P. , Premkumar, P. , Priyanka, R. , Jayachandran, K. S. , & Anusuyadevi, M. (2015). Pathological changes in hippocampal neuronal circuits underlie age‐ associated neurodegeneration and memory loss: Positive clue toward SAD. Neuroscience, 301, 90–105. 10.1016/j.neuroscience.2015.05.062 26045180

[brb31093-bib-0045] Mukherjee, S. , & Manahan‐Vaughan, D. (2013). Role of metabotropic glutamate receptors in persistent forms of hippocampal plasticity and learning. Neuropharmacology, 66, 65–81. 10.1016/j.neuropharm.2012.06.005 22743159

[brb31093-bib-0046] Nasir, M. N. , Abdullah, J. , Habsah, M. , Ghani, R. I. , & Rammes, G. (2012). Inhibitory effect of asiatic acid on acetylcholinesterase, excitatory post synaptic potential and locomotor activity. Phytomedicine, 19(3–4), 311–316. 10.1016/j.phymed.2011.10.004 22112723

[brb31093-bib-0047] Nasir, M. N. , Habsah, M. , Adzim, M. K. R. , Norhayati, A. H. , Muralidhara, D. V. , & Zubaidi, A. L. (2015). Acute effects of triterpene compounds on locomotor performance and Morris water maze tasks in Spraque‐Dawley rats. Biomedical Research, 26(2), 304–310.

[brb31093-bib-0049] Nuss, P. (2015). Anxiety disorders and GABA neurotransmission: A disturbance of modulation. Neuropsychiatric Disease and Treatment., 2015(11), 165–175. 10.2147/NDT.S58841 PMC4303399 25653526

[brb31093-bib-0050] O'Neill, P. K. , Gordon, J. A. , & Sigurdsson, T. (2013). Theta oscillations in the medial prefrontal cortex are modulated by spatial working memory and synchronize with the hippocampus through its ventral subregion. Journal of Neuroscience, 33(35), 1421114224. 10.1523/JNEUROSCI.2378-13.2013 PMC3756763 23986255

[brb31093-bib-0051] Orhan, I. E. (2012). *Centella asiatica* (L.) urban: From traditional medicine to modern medicine with neuroprotective potential. Evidence‐Based Complementary and Alternative Medicine, 2012, 1–8. 10.1155/2012/946259 PMC3359802 22666298

[brb31093-bib-0052] Pandey, S. P. , Singh, H. K. , & Prasad, S. (2015). Alterations in hippocampal oxidative stress, expression of AMPA receptor GluR2 subunit and associated spatial memory loss by Bacopa monnieri extract (CDRI‐08) in streptozotocin‐induced diabetes mellitus type 2 mice. PLoS ONE, 10(7), e0131862 10.1371/journal.pone.0131862 26161865 PMC4498885

[brb31093-bib-0053] Prakash, A. , & Kumar, A. (2013). Mitoprotective effect of *Centella asiatica* against aluminuminduced neurotoxicity in rats: Possible relevance to its anti‐oxidant and anti‐apoptosis mechanism. Neurological Sciences, 34(8), 1403–1409. 10.1007/s10072-012-1252-1 23224641

[brb31093-bib-0054] Rahman, M. M. , Sayeed, M. S. B. , Haque, M. A. , Hassan, M. M. , & Islam, S. A. (2012). Phytochemical screening, antioxidant, anti‐alzheimer and antidiabetic activities of *Centella asiatica* . Journal of Natural Product and Plant Resources, 2(4), 504–511.

[brb31093-bib-0055] Rao, S. B. , Chetana, M. , & Devi, P. U. (2005). *Centella asiatica* treatment during postnatal period enhances learning and memory in mice. Physiology & Behavior, 86(4), 449457. 10.1016/j.physbeh.2005.07.019 16214185

[brb31093-bib-0056] Rao, K. M. , Rao, M. S. , & Rao, G. S. (2012). Evaluation of amygdaloid neuronal dendritic arborization enhancing effect of *Centella asiatica* (Linn) fresh leaf extract in adult rats. Chinese Journal of Integrative Medicine, 18, 1–6. 10.1007/s11655-012-1235-3 23212568

[brb31093-bib-0058] Robert, A. , & Howe, J. R. (2003). How AMPA receptor desensitization depends on receptor occupancy. Journal of Neuroscience, 23(3), 847–858. 10.1523/JNEUROSCI.23-03-00847.2003 12574413 PMC6741906

[brb31093-bib-0059] Salihu, A. T. , Muthuraju, S. , Yusoff, A. A. M. , Ahmad, F. , Mustafa, M. Z. , Jaafar, H. , … Abdullah, J. M. (2016). Mouse model of intracerebellar haemorrhage. Behavioural Brain Research, 312, 374–384. 10.1016/j.bbr.2016.06.034 27327104

[brb31093-bib-0060] Sanderson, D. J. , & Bannerman, D. M. (2012). The role of habituation in hippocampusdependent spatial working memory tasks: Evidence from GluA1 AMPA receptor subunit knockout mice. Hippocampus, 22(5), 981–994. 10.1002/hipo.20896 21125585 PMC3490380

[brb31093-bib-0061] Saoji, S. D. , Raut, N. A. , Dhore, P. W. , Borkar, C. D. , Popielarczyk, M. , & Dave, V. S. (2016). Preparation and evaluation of phospholipid‐based complex of standardized *centella extract* (SCE) for the enhanced delivery of phytoconstituents. The AAPS Journal, 18(1), 102–114. 10.1208/s12248-015-9837-2 26563253 PMC7583548

[brb31093-bib-0062] Sari, D. C. R. , Aswin, S. , Susilowati, R. , Ar‐Rochmah, M. , Prakosa, D. , Romi, M. , & Arfian, N. (2014). Ethanol extracts of *Centella asiatica* leaf improves memory performance in rats after chronic stress via reducing nitric oxide and increasing brain‐derived neurotrophic factor (BDNF) concentration. Journal of Psychology, 1(1).

[brb31093-bib-0063] Schneider, M. (2013). Adolescence as a vulnerable period to alter rodent behavior. Cell and Tissue Research, 354(1), 99–106. 10.1007/s00441-013-1581-2 23430475

[brb31093-bib-0064] Sharma, S. , & Gupta, G. L. (2016). Effect of hydroalcoholic extract of *Centella asiatica* and its synergy with n– acetyl cysteine on marble–burying behavior in mice: Implications for obsessive–compulsive disorder. Austin Journal of Pharmacology and Therapeutics, 4(2), 1083.

[brb31093-bib-0065] Shen, H. , Sabaliauskas, N. , Yang, L. , Aoki, C. , & Smith, S. S. (2017). Role of α4‐containing GABA A receptors in limiting synaptic plasticity and spatial learning of female mice during the pubertal period. Brain Research, 1654, 116–122. 10.1016/j.brainres.2016.01.020 26826007 PMC4959998

[brb31093-bib-0066] Sirichoat, A. , Chaijaroonkhanarak, W. , Prachaney, P. , Pannangrong, W. , Leksomboon, R. , Chaichun, A. , … Welbat, J. U. (2015). Effects of asiatic acid on spatial working memory and cell proliferation in the adult rat hippocampus. Nutrients, 7(10), 84138423. 10.3390/nu7105401 PMC4632421 26445061

[brb31093-bib-0067] Spellman, T. , Rigotti, M. , Ahmari, S. E. , Fusi, S. , Gogos, J. A. , & Gordon, J. A. (2015). Hippocampal‐prefrontal input supports spatial encoding in working memory. Nature, 522(7556), 309–314. 10.1038/nature14445 26053122 PMC4505751

[brb31093-bib-0068] Thoo, Y. Y. , Abas, F. , Lai, O. M. , Ho, C. W. , Yin, J. , Hedegaard, R. V. , & Tan, C. P. (2013). Antioxidant synergism between ethanolic *Centella asiatica* extracts and α‐tocopherol in model systems. Food Chemistry, 138(2), 1215–1219. 10.1016/j.foodchem.2012.11.013 23411234

[brb31093-bib-0069] Tripathi, G. , Mishra, S. , Upadhyay, P. , Purohit, S. , & Dubey, G. P. (2015). Ethnopharmacological importance of *Centella asiatica* with special reference to neuroprotective activity. Asian Journal of Pharmacology and Toxicology, 3(10), 49–53.

[brb31093-bib-0070] Uysal, N. , Kiray, M. , Sisman, A. R. , Camsari, U. M. , Gencoglu, C. , Baykara, B. , & Aksu, I. (2015). Effects of voluntary and involuntary exercise on cognitive functions, and VEGF and BDNF levels in adolescent rats. Biotechnic & Histochemistry, 90(1), 5568. 10.3109/10520295.2014.946968 25203492

[brb31093-bib-0071] Vasavi, H. S. , Arun, A. B. , & Rekha, P. D. (2016). Anti‐quorum sensing activity of flavonoidrich fraction from *Centella asiatica* L. against Pseudomonas aeruginosa PAO1. Journal of Microbiology, Immunology and Infection, 49(1), 8–15. 10.1016/j.jmii.2014.03.012 24856426

[brb31093-bib-0072] Vorhees, C. V. , & Williams, M. T. (2014). Value of water mazes for assessing spatial and egocentric learning and memory in rodent basic research and regulatory studies. Neurotoxicology and Teratology, 45, 75–90. 10.1016/j.ntt.2014.07.003 25116937

[brb31093-bib-0073] Wang, D. S. , Zurek, A. A. , Lecker, I. , Yu, J. , Abramian, A. M. , Avramescu, S. , & Orser, B. A. (2012). Memory deficits induced by inflammation are regulated by α5subunitcontaining GABA A receptors. Cell Reports, 2(3), 488–496. 10.1016/j.celrep.2012.08.022 22999935 PMC4391624

[brb31093-bib-0075] Wu, C. , & Sun, D. (2015). GABA receptors in brain development, function, and injury. Metabolic Brain Disease, 30(2), 367–379. 10.1007/s11011-014-9560-1 24820774 PMC4231020

[brb31093-bib-0076] Yolanda, D. A. , Sari, D. C. R. , Rochmah, M. A. , & Suharmi, S. (2015). The dose variations effect of *Centella Asiatica* ethanol extract on escape latency's distance morris water maze after chronic electrical stress. Kne Life Sciences, 2(1), 146–153. https://doi.org/10.18502/kls.v2i1.134

[brb31093-bib-0077] Zhu, H. , Pleil, K. E. , Urban, D. J. , Moy, S. S. , Kash, T. L. , & Roth, B. L. (2014). Chemogenetic inactivation of ventral hippocampal glutamatergic neurons disrupts consolidation of contextual fear memory. Neuropsychopharmacology, 39(8), 18801892. 10.1038/npp.2014.35 PMC4059896 24525710

